# The SNP rs516946 Interacted in the Association of MetS with Dietary Iron among Chinese Males but Not Females

**DOI:** 10.3390/nu14102024

**Published:** 2022-05-12

**Authors:** Zhenni Zhu, Zhengyuan Wang, Jiajie Zang, Ye Lu, Ziyi Xiao, Guangyong Zheng, Fan Wu

**Affiliations:** 1 Division of Health Risk Factors Monitoring and Control, Shanghai Municipal Center for Disease Control and Prevention, 1380 West Zhongshan Road, Shanghai 200336, China; zhuzhenni@scdc.sh.cn (Z.Z.); wangzhengyuan@scdc.sh.cn (Z.W.); zangjiajie@scdc.sh.cn (J.Z.); luye@scdc.sh.cn (Y.L.); 2 Department of Social Science, New York University Shanghai, Shanghai 200122, China; zx793@nyu.edu; 3 Bio-Med Big Data Center, Shanghai Institute of Nutrition and Health, Chinese Academy of Sciences, Shanghai 200031, China; 4 Shanghai Medical College, Fudan University, 130 Dongan Road, Shanghai 200032, China

**Keywords:** metabolic syndrome, dietary iron, single nucleotide polymorphism, interaction, sex differences

## Abstract

This study aimed to explore the role of the single nucleotide polymorphism (SNP) rs516946 of the Ankyrin 1 (ANK1) gene in the relationship between dietary iron and metabolic syndrome (MetS) in the Chinese population. A total of 2766 Chinese adults (1284 males and 1482 females) were recruited. A 3-day 24-h dietary recall and weighing of household condiments were used to assess dietary intake. Anthropometric and laboratory measurements were obtained. After adjusting for age, region, years of education, intentional physical exercise, physical activity level, smoking, alcohol use and energy intake, dietary iron and the SNP rs516946 were both correlated with MetS risk and interacted among the male participants. The trend between dietary iron and MetS risk remained among T allele non-carriers of males but not among T allele carriers of males. Both the SNP rs516946 and the ferritin level correlated positively with the aspartate aminotransferase (AST) level. ANK1 SNP rs516946 interacted in the association of MetS with dietary iron among Chinese males while no association was found among females. Periodic blood loss might prevent females from these associations. The SNP rs516946 might correlate with liver function.

## 1. Introduction

Metabolic syndrome (MetS) is a constellation of risk factors for cardiovascular disease and metabolic disease [1]. According to the US National Cholesterol Education Program Adult Treatment Panel III (NCEP-ATP III) definition, a MetS diagnosis can be made if three or more out of the following five criteria are met: (i) elevated blood pressure; (ii) elevated waist circumference; (iii) elevated fasting glucose; (iv) elevated triglycerides; (v) reduced high-density lipoprotein cholesterol level [2]. An increase in the prevalence of MetS among adults in China has been observed over the years, and studies have been carried out to examine risk factors for the disease [3,4,5].

Previous studies have shown the positive association of dietary iron intake with MetS [5]. Iron overload has been confirmed as a risk factor of type 2 diabetes mellitus (T2DM) [5,6,7]. The ANK1 gene is located in the 8p11.1 region of the human chromosome, serving as the link between the integral membrane skeleton and the plasma membrane in the red cell [8]. Specifically, the mutation of the SNP rs516946 in the ANK1 gene has been identified as being associated with iron overload, which plays a role in the progression of T2DM, and, thus, the site is regarded as a T2DM-susceptibility locus [9,10]. Similarly, a frameshift mutation of ANK1 has been found in patients with body iron overload [11]. Experiments have suggested that SNPs can induce iron overload caused by the low expression of hepcidin, which is responsible for the liver-specific disruption of Smad4. Moreover, the disturbance of iron in the metabolism will lead to iron being preternaturally deposited in the liver and other parenchyma, and will then induce tissue damage or subclinical inflammation [5,10,12,13]. It has been reported that serum ferritin level is positively correlated with insulin resistance (IR), specifically, dysmetabolic iron overload syndrome (DIOS) can often be detected in patients with fatty liver disease where excess fatty acids accumulate in the liver and induce insulin resistance (IR), which is thought to be a key factor in MetS development [14,15,16]. 

In this study, we aimed to explore the role of the rs516946 site of the gene ANK1 in the relationship between dietary iron and MetS, and to hypothesize the possible underlying mechanism for the site in the development of MetS among adults in China. 

## 2. Materials and Methods

### 2.1. Study Population

**Figure 1:**
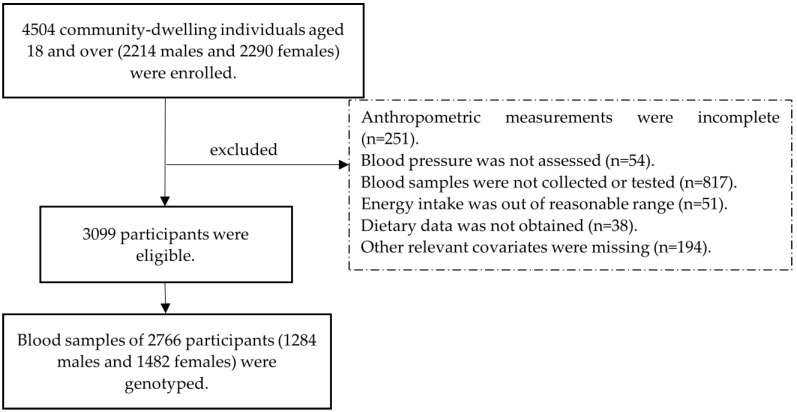
Flow chart of the study participants.

Initially, a total of 4504 community-dwelling individuals aged over 18 years old (2214 males and 2290 females) were selected by random sampling from communities in Shanghai, China from 2012 to 2013. In the current analysis, the participants with lack of anthropometric measurements (*n* = 251), missing blood pressure assessment (*n* = 54), uncollected or untested blood samples (*n* = 817), energy intake less than 300 kcal or more than 3500 kcal daily (*n* = 51), without dietary data (*n* = 38) or other relevant covariates (*n* = 194) were excluded. Among the 3099 eligible participants, the SNP rs516946 of the gene ANK1 was genotyped in 2766 participants (1284 males and 1482 females) (Figure 1).

The Shanghai Municipal Center for Disease Control and Prevention’s institutional review board had approved this study on the 1 January 2012, and the study was carried out in accordance with the code of ethics of the World Medical Association (Declaration of Helsinki). Informed consent was obtained from all participants before the survey.

### 2.2. Dietary Assessment

A 3-day 24-h dietary recall method (2 weekdays and 1 weekend day) was used to collect dietary intake. The weights of some household condiments rich in fat or sodium were collected at the beginning and at the end of the 3 survey days. The public health practitioners from local community health service centers were responsible for investigation. The participants were instructed to record their daily dietary intake at home as well as out of home on draft paper. The public health practitioners conducted face-to-face interviews with participants in the consecutive survey days at home. They first checked through the draft papers, and then revised the food weight and transcribed the draft dietary information into a structured form. The participants were required not to change their daily diet and physical activity patterns during the survey. Nutrition experts from the local Centers for Disease Control and Prevention provided the final review of the structured diet records.

Daily food and condiment consumption was calculated from the 3-day 24-h dietary recall and household condiments weighing. The Chinese food composition database was used to estimate the intake of dietary energy, macronutrients and iron according to daily food and condiment consumption [17,18]. Salt was the only general food that was compulsorily fortified with iodine in China. Only 0.7% of the Chinese population were consuming nutrient supplements and not all the supplements contained the element of iron [19], so dietary supplements were not taken into dietary iron account.

### 2.3. Potential Confounders

The following potential confounders were obtained: age and sex; region (central city, border area and outskirts); years of education (years receiving education); physical activity level (categorized as sedentary, moderate and vigorous); intentional physical exercise (who went for physical exercise at least 20 min per day for purpose); smoking (never, former and current); alcohol use (four categories including lifetime abstainers, nonheavy drinkers/social drinkers, infrequent heavy drinkers/binge drinkers and frequent heavy drinkers.

### 2.4. Anthropometric and Laboratory Measurements

The anthropometric measurements were performed in each participant’s community at the local health center. A Graham-Field 1340-2 tape measure was used to measure waist circumference. An Omron HEM-7071 electronic sphygmomanometer (Omron Healthcare, Kyoto, Japan) was used to assess resting blood pressure.

Fasting venous blood samples were collected to measure several indicators. A HITACHI 7080 Automatic Biochemical Analyzer with reagents from Wako Pure Chemical Industries, Ltd. (Tokyo, Japan) was used to analyze glucose, triglycerides, high-density lipoprotein cholesterol (HDL-C) and aspartate aminotransferase (AST). A Chemiluminescence Immune Detection System (ACCESS 2, Beckman Coulter, Los Angeles, CA, USA) was used to measure ferritin. All the above indicators were measured during 2012–2013 at the laboratory of Shanghai Municipal Center for Disease Control and Prevention. 

The white blood cells were stored at −80℃ temperature just after collection during the 2012–2013 fieldwork. DNA was first extracted from separated white blood cells using the magnetic bead method by the Universal Genomic DNA Extraction Kit (type DP705-02 from TIANGEN, Beijing, China) in 2018. Then, the SNP rs516946 was amplified and genotyped using a SNaPshot Multiplex System on a genetic inheritance analyzer (type 3730XL from Applied Biosystems, Waltham, MA, USA). The electropherograms were analyzed using GeneMapper software.

### 2.5. Definition of Metabolic Syndrome

According to the criteria of the US National Cholesterol Education Program Adult Treatment Panel III (NCEP-ATP III) for Asian populations [20], MetS was defined as the presence of three or more of the following metabolic abnormalities: (1) elevated waist circumference (WC ≥ 90 cm for men or ≥80 cm for women); (2) elevated triglycerides (triglycerides ≥ 150 mg/dL) or using drugs treating hypertriglyceridemia; (3) reduced HDL-C (HDL-C < 40 mg/dL for men or <50 mg/dL for women) or on drug treatment for reduced HDL-C; (4) elevated blood pressure (systolic blood pressure ≥ 130 mmHg and/or diastolic blood pressure ≥ 85 mmHg) or using drugs treating hypertension; (5) elevated fasting glucose (fasting glucose ≥ 100 mg/dL) or on drug treatment for hyperglycemia.

### 2.6. Statistical Analysis

The odds ratios (ORs) and 95% confidence intervals (CIs) of MetS were analyzed using logistic regression models, where the occurrence of MetS (or its components) and the quartiles of dietary iron intake were treated as the dependent variables and the independent variables, respectively. In the same logistic regression model, a product term of T allele presence of rs516946 (a binary variable coded as 1 for presence and 0 for non-presence) and iron intake was used to estimate the interaction departure from multiplicativity. Pearson correlation analysis was utilized to determine the relationship between AST and the ferritin level, AST and the SNP rs516946. These statistical analyses were conducted with SAS statistical software (v. 9.4; SAS Institute, Cary, NC, USA).

Logistic regression models were also introduced to estimate the interaction departure from additivity [21], while the bootstrapping method was used to calculate the CIs around the estimate of interaction. Three indices, the Relative Excess Risk due to Interaction (RERI), Attributable Proportion (AP) and Synergy index (S), were measured for interaction on an additive scale. AP indicated the proportion of MetS attributable to the dietary iron and the SNP rs516946 interaction. S meant the ratio between the combined effect and the sum of the individual effects. For the bootstrap method, 10,000 samples (with replacements) were generated. Each sample had the same size as the original sample. The RERI was then estimated in each of these new samples and the 95% CI for RERI was estimated as the 2.5th and 97.5th percentiles of the resulting bootstrap sampling distribution. These statistical analyses were conducted with S-PLUS 6.2 (S-PLUS 6.2, Insightful, Seattle, WC, USA).

## 3. Results

### 3.1. Characteristics of the Participants

Participants’ characteristics are shown in Table 1. A total number of 2766 Chinese adults were included in the study, with 1284 (46.4%) males and 1482 (53.6%) females. The average daily dietary iron intake was 19.7 ± 16.3 mg for all participants, 22.0 ± 20.4 mg for male participants, and 17.7 ± 11.3 mg for female participants. T allele presence on rs516946 was 23.7% in all, 21.9% in male participants, and 25.4% in female participants. The prevalence of MetS was 23.9% in all, 21.8% in male participants and 25.7% in female participants.

### 3.2. The Associations of MetS Risk with Dietary Iron and the SNP rs516946 

After adjusting for age, sex, region, years of education, physical activity level, intentional physical exercise, smoking status, alcohol use and dietary total energy intake, dietary iron was positively associated with MetS risk (*p* < 0.001) but not the SNP rs516946 (*p* = 0.146) in all participants. When stratified by sex, adjusting the same confounders except sex, dietary iron and the SNP rs516946 were both in the linear correlations with MetS risk among the male participants (*p* < 0.001 and = 0.019). Furthermore, the multiplicative interaction between dietary iron and the SNP rs516946 was also found among the male participants (*p* = 0.007). However, no association of MetS risk was found with dietary iron or the SNP rs516946 in the female participants (*p* = 0.121 and = 0.796). Neither was there a multiplicative interaction between dietary iron and the SNP rs516946 in the female participants. (Table 2 and Table 3).

Regarding the multiplicative interaction between dietary iron and the SNP rs516946 among the male participants, additive interaction analysis was further conducted. Significant additive interaction was observed between dietary iron and the SNP rs516946 among male participants. The RERI (95% CI) was −0.81 (−2.23, −0.13) (Table 4). 

### 3.3. The Associations between Dietary Iron and MetS Risk Stratified by T Allele Presence of rs516946

Further analysis was conducted using the data from the male participants, since the associations mentioned above were found only in male participants. T allele on the rs516946 was set to be the risk allele. After adjusting for age, region, years of education, physical activity level, intentional physical exercise, smoking status, alcohol use and dietary energy intake (Model 2), dietary iron was still associated with MetS risk in the T allele non-carriers (*p* < 0.001). However, there was no association in the T allele carriers (*p* = 0.854) and the ORs for MetS risk across the quartiles of the dietary iron intake compared with the reference group (the lowest dietary iron intake subgroup in the T allele non-carriers), which were 1.78, 1.48, 1.97 and 1.81 (Table 5).

**Figure 2:**
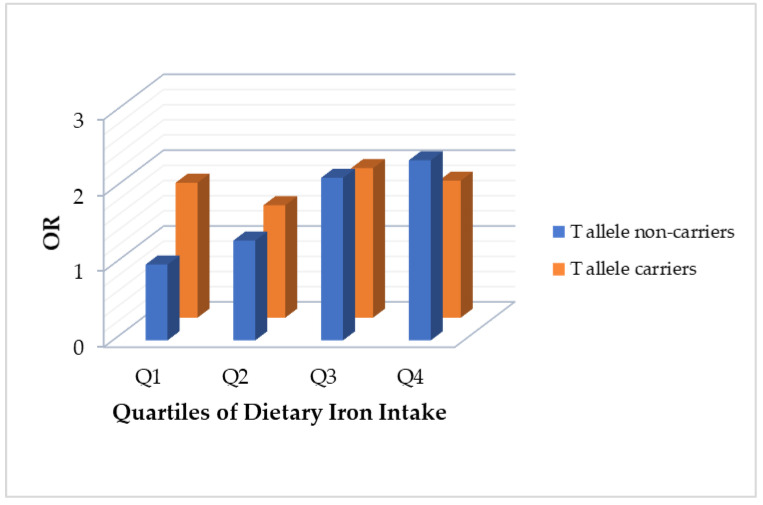
The association between dietary iron intake and MetS risk stratified by the T allele presence of rs516946 among the male participants.

Figure 2 shows the associations between dietary iron and MetS risk among the male participants stratified by T allele presence of rs516946.

### 3.4. The Correlation between Liver Metabolic Indicators and SNP rs516946

The analysis was still conducted using the data from the male participants. After adjusting for the SNP rs516946 and age, a positive correlation between the AST and ferritin level (r = 0.258, *p* < 0.001) was found. After adjusting for the ferritin level and age, a weak positive correlation between the SNP rs516946 and AST was also found (r = 0.049, *p* = 0.068) (Table 6).

## 4. Discussion

In the current study, consistent with the previous findings, dietary iron intake appeared to be positively associated with MetS in the male participants but not in the female participants. Dietary iron is related to the body’s iron storage. The typical Chinese diet consisted mainly of plant food and most of the dietary iron was from nonheme iron. In our previous study, we had found nonheme iron intake was associated with MetS risk in Chinese population [22], which was consistent with those studies on Asian population that nonheme iron contributed to meet body needs when diets were dominant in plant food. Excessive free iron is a strong pro-oxidant and induces oxidative damage and apoptosis in the body [23]. The side effects of iron are balanced out through binding to ferritin, an iron storage protein [24]. The average serum ferritin level was within the reasonable range in the current study, but a recent publication indicated that even when ferritin was in the clinically normal range, a dose–response association of MetS risk always occurred [25]. We found the association between the SNP rs516946 and MetS in the male participants but still not in the female participants. Periodic blood loss might play a protective role in women that kept them from excessive iron accumulation in the body [26]; moreover, since women are more likely to suffer from iron deficiency instead, iron therapy could even help with menstruating women’s health [27]. Therefore, it is less likely to find the association between dietary iron and MetS in women [22]. Excessive iron in the body might be the crucial linkage to abnormal metabolism overall. 

Furthermore, we discovered the SNP rs516946 was not only significantly associated with MetS but interacted with dietary iron intake in the male participants. We assumed that the T allele of rs516946 was the risk allele. Among T allele non-carriers of males, the linear trend of dietary iron intake positively correlated with MetS, while for T allele carriers of males, such increased trend association, was no longer observed and the risk of MetS was at a higher level, overall, compared with the reference group (the lowest dietary iron intake subgroup in the T allele non-carriers). Is it possible that the rs516946 risk allele T might be associated with iron dysmetabolism, and thus, no matter how low the dietary iron intake, the disturbance of iron in the metabolism could always cause iron overload in the liver, which might lead to MetS? 

In order to assess the relationship between liver function and iron load possibly caused by carrying the rs516946 risk allele, an association evaluation between the AST level and ferritin level and the SNP rs516946 was carried out. It showed that the ferritin level was positively associated with the AST level, which coincided with the previous studies showing that the level of iron in the body was linked with liver function [28]. The SNP rs516946 presented a weak positive association with the AST level in the current study. Some studies show that iron loads correlate with liver function [14,22]. It was hypothesized that the SNP rs516946 might correlate with liver function mediated by influencing iron regulation.

A limitation of this study is the assessment of dietary intake. Three-day 24-h dietary recalls were used to obtain food consumption, so the information on dietary intake might not be accurate since the participants depended on their recall and estimations. Moreover, non-response bias might occur, and the results for the risk factor analysis might be influenced by other unknown confounding factors. Moreover, only the data on ferritin was available in the current study; other serum indicators such as free iron or transferrin were not obtained. This might cause bias to assess the body’s iron load. Furthermore, when stratified by the male participants carrying or not carrying the risk allele, the sample size in each subgroup was likely not enough to reach a significant statistical result of OR. This might cause bias in the interpretation of the results. In addition, the current study focused on the SNP instead of the haplotype. Bias might exist in the current results for the associations between the locus polymorphism and risk of MetS in the male participants. Finally, the SNP might influence the body’s metabolism, but we cannot conclude the causal inferences between ANK1 SNP rs516946 and MetS for the cross-sectional nature of this study.

## 5. Conclusions

ANK1 SNP rs516946 interacted in the association between dietary iron and MetS among Chinese males while no association was found among females. Periodic blood loss might prevent females from these associations. The SNP rs516946 might correlate with liver function. 

## Figures and Tables

**Table 1:** Characteristics of the participants by sex.

	All	Male	Female	*p*
*n* (%)	2766 (100.0)	1284 (46.4)	1482 (53.6)	
Age (%)				0.513
18–44 years	30.8	30.1	31.4	
45–59 years	36.2	36.3	36.1	
≥60 years	33.0	33.6	32.6	
Years of Education, years (SD ^1^)	9.5 (4.5)	10.2 (4.0)	8.9 (4.9)	<0.001
Physical Activity Level (%)				<0.001
Sedentary	84.2	78.7	89.0	
Moderate	13.8	18.1	10.2	
Vigorous	2.0	3.3	0.8	
Intentional Physical Exercise (%)	24.8	25.2	24.5	0.94
Smoking Status, %				
Never smoked	72.0	40.8	98.8	
Former smoker	5.0	10.5	0.3	
Current smoker	23.0	48.8	1.0	
Alcohol use (%)				<0.001
Lifetime abstainers	80.7	64.0	94.9	
Nonheavy drinkers	15.1	27.3	4.6	
Infrequent heavy drinkers	1.3	2.6	0.3	
Frequent heavy drinkers	3.0	6.1	0.3	
Dietary Intake				
Energy, kcal/day (SD)	1760.9 (842.5)	1938.5 (884.5)	1608.9 (773.4)	<0.001
Total iron, mg/day (SD)	19.7 (16.3)	22.0 (20.4)	17.7 (11.3)	<0.001
Heme iron, mg/day (SD)	1.6 (1.5)	1.7 (1.6)	1.5 (1.4)	<0.001
Nonheme iron, mg/day (SD)	18.4 (16.6)	20.7 (21.0)	16.3 (10.7)	<0.001
Ferritin, ng/mL (SD)	124.5 (122.5)	165.8 (139.2)	87.0 (89.8)	<0.001
Metabolic Syndrome (%)	23.9	21.8	25.7	0.011
Metabolic Syndrome Components				
Elevated blood pressure (%)	52.4	56.8	48.7	<0.001
Elevated waist circumference (%)	33.6	25.5	40.5	<0.001
Elevated fasting glucose (%)	23.1	25.4	21.1	0.007
Elevated triglycerides (%)	27.2	30.3	24.4	<0.001
Reduced HDL-C ^2^, %	21.0	14.8	26.2	<0.001
T allele presence on rs516946 (%)	23.7	21.9	25.4	<0.001
Genotypes of rs516946 (%)				0.079
CC ^3^	76.3	78.1	74.7	
CT ^4^	22.4	20.8	23.9	
TT ^5^	1.3	1.1	1.4	

^1^ SD, standard deviation; ^2^ HDL-C, high-density lipoprotein cholesterol; ^3^ CC, double C allele; ^4^ CT, one C allele and one T allele; ^5^ TT, double T allele.

**Table 2:** Logistic regression results for MetS risk according to dietary iron and SNP rs516946 in the participants stratified by sex ^1^.

	*β*	*p*
All		
Dietary iron	0.21	<0.001
rs516946	0.38	0.146
Male		
Dietary iron	0.34	<0.001
rs516946	1.02	0.019
Female		
Dietary iron	0.12	0.121
rs516946	0.08	0.796

^1^ T allele presence of rs516946 was coded as 1 for presence and 0 for non-presence.

**Table 3:** Multiplicative interaction results between dietary iron intake and SNP rs516946 among participants ^1^.

	*β*	*p*
All	−0.21	0.033
Male	−0.43	0.007
Female	−0.11	0.381

^1^ T allele presence of rs516946 was coded as 1 for presence and 0 for non-presence.

**Table 4:** Additive interaction results (95% CI) for dietary iron intake and the SNP rs516946 among the male participants ^1^.

Gene Site	RERI ^2^	AP ^3^	S ^4^
rs516946	–0·81 (–2.23, –0.13)	–0.31 (–0.58, –0.08)	0.70 (0.51, 0.86)

^1^ T allele presence of rs516946 was coded as 1 for presence and 0 for non-presence. ^2^ RERI, Relative Excess Risk due to Interaction. ^3^ AP, Attributable Proportion; ^4^ S, Synergy index.

**Table 5:** ORs (95% CI) for MetS and its components according to the quartiles of total dietary iron intake among the male participants stratified by T allele presence of rs516946 ^1^.

		Q1	Q2	Q3	Q4	*p*-Value for Trend ^2^
Total Iron Intake (mg/day)		<14.17	(14.17–17.91)	(17.91–23.87)	≥23.87	
*n*		352	351	353	351	
Metabolic syndrome						
Model 1 ^3^	T allele non-carriers	Reference	1.36 (0.85, 2.16)	2.06 (1.32, 3.22)	2.14 (1.38, 3.34)	<0.001
	T allele carriers	1.92 (0.99, 3.76)	1.46 (0.63, 3.39)	1.62 (0.76, 3.48)	1.01 (0.41, 2.45)	0.218
Model 2 ^4^	T allele non-carriers	Reference	1.32 (0.81, 2.15)	2.14 (1.31, 3.52)	2.37 (1.36, 4.14)	<0.001
	T allele carriers	1.78 (0.87, 3.63)	1.48 (0.58, 3.78)	1.97 (0.73, 5.32)	1.81 (0.52, 6.32)	0.854
Metabolic syndrome clinical indexes						
Elevated blood pressure						
Model 1	T allele non-carriers	Reference	1.24 (0.85, 1.83)	1.17 (0.79, 1.72)	1.32 (0.90, 1.95)	0.217
	T allele carriers	1.29 (0.67, 2.47)	1.34 (0.62, 2.88)	1.21 (0.59, 2.48)	1.11 (0.51, 2.39)	0.643
Model 2	T allele non-carriers	Reference	1.28 (0.85, 1.92)	1.34 (0.87, 2.06)	1.67 (1.02, 2.74)	0.052
	T allele carriers	1.12 (0.57, 2.21)	1.33 (0.58, 3.02)	1.23 (0.54, 2.81)	1.37 (0.50, 3.81)	0.757
Elevated waist circumference						
Model 1	T allele non-carriers	Reference	1.52 (0.99, 2.34)	1.86 (1.22, 2.84)	2.07 (1.36, 3.15)	<0.001
	T allele carriers	1.57 (0.82, 3.03)	1.60 (0.72, 3.56)	1.65 (0.79, 3.45)	1.76 (0.80, 3.88)	0.773
Model 2	T allele non-carriers	Reference	1.42 (0.91, 2.23)	1.68 (1.07, 2.66)	1.74 (1.04, 2.90)	0.028
	T allele carriers	1.45 (0.72, 2.92)	1.66 (0.69, 3.97)	2.27 (0.91, 5.63)	3.03 (1.01, 9.14)	0.166
Elevated fasting glucose						
Model 1	T allele non-carriers	Reference	1.02 (0.67, 1.54)	1.14 (0.76, 1.72)	1.36 (0.91, 2.03)	0.112
	T allele carriers	0.66 (0.32, 1.35)	1.12 (0.46, 2.70)	1.19 (0.52, 2.69)	1.11 (0.47, 2.66)	0.244
Model 2	T allele non-carriers	Reference	1.13 (0.73, 1.75)	1.34 (0.85, 2.13)	1.75 (1.04, 2.93)	0.032
	T allele carriers	0.50 (0.22, 1.11)	1.02 (0.39, 2.70)	1.30 (0.48, 3.54)	1.19 (0.36, 3.95)	0.141
Elevated triglycerides						
Model 1	T allele non-carriers	Reference	1.44 (0.98, 2.12)	1.58 (1.07, 2.33)	1.60 (1.09, 2.36)	0.016
	T allele carriers	1.48 (0.81, 2.71)	0.94 (0.43, 2.08)	1.41 (0.70, 2.84)	1.21 (0.56, 2.59)	0.890
Model 2	T allele non-carriers	Reference	1.42 (0.94, 2.13)	1.53 (1.00, 2.36)	1.63 (1.00, 2.64)	0.049
	T allele carriers	1.62 (0.85, 3.10)	0.95 (0.40, 2.26)	1.70 (0.72, 3.99)	1.65 (0.57, 4.77)	0.695
Reduced HDL-C ^5^						
Model 1	T allele non-carriers	Reference	0.95 (0.58, 1.56)	1.34 (0.83, 2.14)	1.12 (0.69, 1.82)	0.372
	T allele carriers	1.12 (0.52, 2.39)	0.47 (0.15, 1.46)	0.88 (0.35, 2.19)	0.95 (0.36, 2.53)	0.988
Model 2	T allele non-carriers	Reference	0.97 (0.58, 1.64)	1.51 (0.88, 2.57)	1.38 (0.74, 2.56)	0.156
	T allele carriers	1.16 (0.53, 2.57)	0.59 (0.18, 1.97)	1.25 (0.41, 3.83)	1.81 (0.45, 7.26)	0.441

^1^ T allele presence on rs516946 was coded as 1 for presence and 0 for non–presence. ^2^ The *p*–value for the trend was examined using each quartile’s median of dietary iron intake. ^3^ Model 1 was adjusted for age. ^4^ Model 2 was adjusted for age, region, years of education, intentional physical exercise, physical activity level, smoking status, alcohol use and dietary total energy intake. ^5^ HDL-C, high-density lipoprotein cholesterol.

**Table 6:** Pearson Correlation of AST with ferritin level and SNP rs516946 in the male participants ^1^.

	*n*	r ^4^	95% CI	*p*
AST and ferritin level ^2^	1404	0.258	(0.21, 0.31)	<0.001
AST and the SNP rs516946 ^3^	1400	0.049	(0.00, 0.10)	0.068

^1^ T allele presence on rs516946 was coded as 1 for presence and 0 for non–presence. ^2^ The correlation between AST and ferritin level was determined after adjusting for age and SNP rs516946. ^3^ The correlation between AST and SNP rs516946 was determined after adjusting for age and ferritin level. ^4^ Correlation coefficient.

## Data Availability

The datasets used and analyzed in the current study are available from the corresponding author on reasonable request.

## Abbreviations

SNP: single nucleotide polymorphism; MetS, metabolic syndrome; AST, aspartate aminotransferase; T2DM, type 2 diabetes mellitus; IR, insulin resistance; HDL-C, high-density lipoprotein cholesterol; OR, odds ratio; 95% CI, 95% confidence interval; SDHS, Shanghai Diet and Health Survey.
